# Bacteriological profile of conjunctiva bacterial Flora in Northeast China: a hospital-based study

**DOI:** 10.1186/s12886-022-02441-8

**Published:** 2022-05-16

**Authors:** Shuo Xu, Hong Zhang

**Affiliations:** 1 Department of Ophthalmology, The Affiliated Wuxi No. 2 People’s Hospital of Nanjing Medical University, WuXi, Jiangsu Province People’s Republic of China; 2 Eye Hospital, The First Affiliated Hospital of Harbin Medical University, Harbin, 150000 Heilongjiang Province People’s Republic of China

**Keywords:** Conjunctival bacterial flora, Sex, Seasonal changes, Northeastern China

## Abstract

**Purpose:**

To investigate the distribution and influencing factors of preoperative conjunctival flora from patients undergoing penetrating ocular surgeries in northeast China.

**Methods:**

An observational and cross-sectional study design was used. In 305 eyes of 305 patients without infective eye diseases who underwent ocular surgeries at the First Affiliated Hospital of Harbin Medical University between May 2018 and May 2019, conjunctival sac scrapings were collected on the day before surgery.

**Results:**

The mean age of the participants was 60.73 ± 14.23 years, with the majority being female, married and unemployed with at least primary school education. The positive culture rate was 48.20% (147/305 eyes), and 191 bacterial strains were isolated; two or more strains were isolated from 22.45% (33/147) of positive samples. The most commonly isolated bacteria was *Staphylococcus epidermidis* (64.92%), surpassing *Staphylococcus aureus* (5.76%). The culture positive rate of the young (18–40 years) group was different between the females (26.67%) and males (69.23%) (*P* = 0.024), but in the middle-aged group and the elderly group, the rates between the sexes were similar, with an increasing trend. Patients who visited in summer or autumn presented a higher positive rate than other seasons. Hypertensive women had a higher rate than hypertensive men (58.14% vs. 40%, χ^2^ = 5.*8662, P* = 0.0154).

**Conclusions:**

In northeastern China, coagulase-negative *Staphylococcus* were the most common preoperative conjunctival bacteria*.* Hypertensive female patients, elderly patients, or those operated on in the summer and autumn should pay more attention to perioperative treatment.

**Trial registration:**

ChiCTR2100044659.

**Supplementary Information:**

The online version contains supplementary material available at 10.1186/s12886-022-02441-8.

## Introduction

The conjunctiva is the mucous membrane that lines the internal aspect of the eyelids and extends to the orbital globe, and the conjunctival sac is the space bound between the palpebral and bulbar conjunctiva [1]. Given its continuous exposure to the outside environment, normal commensal flora and potential pathogens can be detected in the conjunctival sac [2]. The normal commensal flora can protect the host either by occupying potential colonization sites of pathogens, producing antimicrobial products or stimulating an immune response that protects the host against infection [2]. However, random or surgical penetrating trauma can promote the migration of resident bacterial flora into the eye, leading to endophthalmitis [3, 4]. Therefore, understanding and monitoring the distribution of conjunctival bacteria are important in pre, peri- and postoperative management.

*Coagulase-negative staphylococci* (CoNS) and *Corynebacterium* sp. are the two most commonly found bacterial flora of the normal conjunctiva [5, 6]. Due to climate changes and local dietary habits between the areas, geographic location influences the distribution of conjunctival flora. A previous study compared the conjunctival microbiome among three major Chinese cities (Guangzhou, Wenzhou, and Beijing), thousands of kilometres apart within different climates, and Beijing conjunctival samples displayed elevated diversity and abundance compared to other cities [7]. Numerous causative factors have been linked to changes in the composition of ocular surface flora, including ocular diseases (dry eye [8] and infectious keratitis), systemic diseases (diabetes [9] and hyperlipidaemia [10]), alcoholism [11] and patient age [12]. Investigating the conjunctival flora in specific populations has long been a question of interest in a wide range of fields. Heilongjiang Province is located in northeast China. The weather is dry and cold, and no study of conjunctiva flora in this area has been reported.

The objective of this study was to investigate the bacterial flora of the conjunctival sac in patients scheduled for ocular surgeries and to explore the influencing factors of culture-positive or varied numbers of bacteria in patients before surgery in a city located in northeast China.

## Materials and methods

### Study subjects

This was an observational and cross-sectional study that enrolled 305 eyes from 305 patients undergoing eye surgery at the First Affiliated Hospital of Harbin Medical University between May 2018 and May 2019. The inclusion criteria included consenting patients suffering from cataract, diabetic retinopathy, pterygium, strabismus, macular hole or epimacular membrane, retinal detachment and silicone oil-filled eye. Patients were excluded if they had active ocular inflammation (dry eye, blepharitis, conjunctivitis chronic dacryocystitis, and keratitis), nasolacrimal duct obstruction, ocular trauma, history of contact lens wear, topical or systemic administration of antimicrobial or immunosuppressive agents in the past 3 months or use of eyedrops in either eye within 3 days. All methods were carried out in accordance with research guidelines and regulations. All experimental protocols were approved by the First Affiliated Hospital of Harbin Medical University Ethical Committee (No. 201848) and strictly followed the Helsinki Declaration. All patients provided written informed consent before participating in the study. This study was registered in the Chinese Clinical Trial Registry (registration number is ChiCTR2100044659).

### Specimen collection from the conjunctiva sac

The culture samples were obtained using a previously described method [13]. The specimens were collected from the lower conjunctival sac of surgical eyes without applying a topical anaesthetic, as follows. The periocular skin and eyelid margins were first cleaned with sterile normal saline. The lower lid was pulled with a cotton swab to expose the lower conjunctival fornix. After asking each subject to gaze upwards, a sterilized swab moistened with a drop of sterile normal saline was used to gently wipe the lower fornix conjunctiva from the medial to lateral side. The individuals were asked to not blink during the procedure to avoid touching the lid margins. Then, the samples were immediately inoculated into thioglycolate broth (Oxoid, England).

### Bacterial culture, isolation and identification

Thioglycolate medium was used as the enrichment culture, and specimens were incubated in 5% CO_2_ at 35 °C for 48 h. The bacteria, if any, were immediately streaked onto 5% sheep blood (JinZhang Technology, China) and chocolate agar plates (JinZhang Technology, China) and incubated at 35 °C for another 24 h. The identification of bacterial species was carried out by analytical profile index (API) panels (BioMérieux, France).

### Statistical analysis

Sociodemographic and clinical characteristics for the study population were summarized with frequencies and percentages for categorical variables and mean values and standard deviations for continuous variables. Variance analysis and the chi-square test were used to compare differences in positivity rate. Spearman rank correlation coefficients were calculated between the total bacterial count and patient characteristics. Univariate logistic analysis was conducted to analyse the relationship between the positive rate and patient characteristics. All statistical analyses were performed using SPSS software (v20.0, SPSS Inc., Chicago, IL, USA). *P* < 0.05 was considered a significant difference.

## Results

### Baseline characteristics

Overall, 305 conjunctival samples were collected from the operative eyes of 305 patients enrolled in the study. The average participant age was 60.73 years ±14.23 SD years (range 18 to 89). The positive culture rate from patients was 48.20% (147/305 eyes). Other sociodemographic and clinical characteristics of the patients are summarized in Table 1.

**Table 1:** Demographic and clinical characteristics of the enrolled patients (*n* = 305)

	Culture-negative patients	Culture-positive patients	Overall	*F/x*^*2*^	*P*
**Patients, n**	158	147	305		
**Age, year**					
mean ± SD	59.92 ± 15.05	61.60 ± 13.27	60.73 ± 14.23	1.06	0.3032
n(%)					
≤ 40	15(9.49)	13(8.84)	28(9.18)	2.4957	0.2871
40–65	87(55.06)	69(46.94)	156(51.15)		
≥ 65	56(35.44)	65(44.22)	121(39.67)		
**Sex, n(%)**					
Female	91(57.59)	87(59.18)	178(58.36)	0.0791	0.7785
Male	67(42.41)	60(40.82)	127(41.64)		
**Education, n(%)**					
No formal education or Primary	38(24.05)	47(31.97)	85(27.87)	2.5171	0.2841
Secondary	69(43.67)	60(40.82)	129(42.30)		
Tertiary	51(32.28)	40(27.21)	91(29.84)		
**Job, n(%)**					
Worker	20(12.66)	30(20.41)	50(16.39)	4.2992	0.2309
Farmer	30(18.99)	30(20.41)	60(19.67)		
Clerk	24(15.19)	16(10.88)	40(13.11)		
Unemployed	84(53.16)	71(48.30)	155(50.82)		
**Marriage, n(%)**					
Married	26(16.46)	24(16.33)	50(16.39)	0.0009	0.9757
Single or Widowed	132(83.54)	123(83.67)	255(83.61)		
**High Blood Pressure, n(%)**					
No	94(59.49)	80(54.42)	174(57.05)	1.4945	0.4737
<10 years	39(24.68)	36(24.49)	75(24.59)		
≥ 10 years	25(15.82)	31(21.09)	56(18.36)		
**Diabetes Mellitus, n(%)**					
No	103(65.19)	101(68.71)	204(66.89)	0.4336	0.8051
<10 years	27(17.09)	23(15.65)	50(16.39)		
≥ 10 years	28(17.72)	23(15.65)	51(16.72)		
**Smoking, n(%)**					
No	123(77.85)	107(72.79)	230(75.41)	1.2735	0.529
<20 years	13(8.23)	17(11.56)	30(9.84)		
≥ 20 years	22(13.92)	23(15.65)	45(14.75)		
**Drinking, n(%)**					
No	134(84.81)	125(85.03)	259(84.92)	0.003	0.9565
Yes	24(15.19)	22(14.97)	46(15.08)		

### Isolation rate and bacterial isolates

The positive culture rate from patients was 48.20% (147/305 eyes). Of the 147 samples with positive culture results, 33 (22.45%) had mixed cultures with two to three isolated strains. Altogether, 31 types of bacteria were identified. The most common microorganism was *Staphylococcus epidermidis* (64.92%), followed by *Staphylococcus aureus* (5.76%) and *Staphylococcus xylosus* (5.24%). The isolation rate of gram-negative bacteria was 8.38%, and the three most common isolates were *Citrobacter freundii* (1.57%), *Burkholderia cepacia* (1.57%) and *Pseudomonas aeruginosa* (1.05%). The microorganism profiles isolated from the conjunctival sac are shown in Table 2.

**Table 2:** Bacterial isolates from the conjunctiva of patients (*n* = 191)

Bacteria	No.isolates	Percentage(%)
**Gram-positive**	175	91.62%
*Staphylococcus epidermidis*	124	64.92%
*Staphylococcus aureus*	11	5.76%
*Staphylococcus xylosus*	10	5.24%
*Corynebacterium species*	7	3.66%
*Klebsiella oxytoca*	3	1.57%
*Staphylococcus lugdunensis*	3	1.57%
*Staphylococcus sciuri*	2	1.05%
*Staphylococcus lentus*	2	1.05%
*Bacillus cereus*	2	1.05%
Others (*Staphylococcus hominis, Enterococcus faecalis, Kocuria roseus, Staphylococcus cohnii urealyticum, Staphylococcus warneri, Rhodococcus, Staphylococcus caprae, Micrococcus luteus, Staphylococcus capitis, Kocuria varians, Bacillus halmapalus*)	11	5.75%
**Gram-negative**	16	8.38%
*Citrobacter freundii*	3	1.57%
*Burkholderia cepacia*	3	1.57%
*Pseudomonas aeruginosa*	2	1.05%
Others (*Enterobacter aerogenes, Delftia acidovorans, Brevendimonas vesicularis, Proteus, Brevendimonas diminuta, Stenotrophomonas maltophilia, Enterobacter cloacae, Mannheimia haemolytica*)	8	4.19%

### Correlation between the culture-positivity rate and clinical factors

First, the separate impacts of age and sex on the culture-positivity rate were analysed. As shown in Table 3, the frequency of culture-positive conjunctiva in females was higher than that in males (48.88% vs. 47.24%, χ^2^ = 0.0791, *P* = 0.7785). According to age, the patients were divided into three groups: 18–40 years old (young), 41–65 years old (middle aged), and > 65 years old (elderly). The positive culture rates were 46.43% (13/28), 44.23% (69/156) and 53.72% (65/121) in each group, respectively. Although not statistically significant (χ^2^ = 2.4957*, P* = 0.2871), the frequency of culture positivity in the conjunctiva of elderly patients was higher than that in young patients.

**Table 3:** Bacterial frequency in different age groups and sex groups

	Sex		*x*^*2*^	*P*	Age			*x*^*2*^	*P*
	Female	Male			≤40	40–65	> 65		
Culture-negative	91(51.12)	67(52.76)	0.0791	0.7785	15(53.57)	87(55.77)	56(46.28)	2.4957	0.2871
Culture-positive	87(48.88)	60(47.24)			13(46.43)	69(44.23)	65(53.72)		

Furthermore, we analysed the interactive impact of age and sex on culture-positivity rates. As shown in Table 4, in the young (18–40 years) group, the frequency of culture positivity in the conjunctiva in male patients (69.23%) was much higher than that in female patients (26.67%) (χ^2^ = 5.072, *P* = 0.024). Between the young group and the middle-aged group, the frequency of culture positivity in the conjunctiva of male patients showed a trend towards an initial reduction, and the incidence then increased with advancing age in the elderly group (Additional file 1). However, the results were completely different for female patients; the frequency of culture positivity in the conjunctiva of female patients increased significantly with age, and the isolation rate of the elderly group (55.41%) was higher than that of the other groups (55.41% vs. 47.19% vs. 26.67%, χ^2^ = 4.3247, *P* = 0.1151). Therefore, age and sex have a synergistic effect on the bacterial isolation of patients.

**Table 4:** The isolation rates of conjunctival samples in each group

Age, year	Age ≤ 40, n(%)		*x*^*2*^	*P*	Age = 40–65, n(%)		*x*^*2*^	*P*	Age > 65, n(%)		*x*^*2*^	*P*
	Culture-negative	Culture-positive			Culture-negative	Culture-positive			Culture-negative	Culture-positive		
Female	11(73.33)	4(26.67)	5.072	0.024	47(52.81)	42(47.19)	0.736	0.390	33(44.59)	41(55.41)	0.217	0.640
Male	4(30.77)	9(69.23)			40(59.70)	27(40.30)			23(48.94)	24(51.06)		

Harbin is located in northeastern China, which has a predominantly dry climate and a lower temperature than other parts of China. According to the internationally recognized astronomical seasons, spring is defined as March 21 to June 21, summer as June 21 to September 22, fall as September 22 to December 21, and winter as December 22 to March 20 of the following year [14]. The culture positivity rate was higher in patients who visited in summer (49.15%) and autumn (56.72%) than in those who visited in spring (41.03%) and winter (45.24%) (Additional file 2). Although the difference was not statistically significant (*P* = 0.0605, *OR (95% CI) =* 1.88 (0.97, 3.65)), the difference in the culture positive rate between autumn and spring was still the largest.

In this study, smoking history was available in 75 patients. Although not statistically significant, the culture-positive rate of patients with a smoking history (53.33%) was higher than that of those who never smoked (46.65%) (χ^2^ = 0.8866, *P* = 0.6419). A history of alcohol consumption did not affect the culture-positive rate (χ^2^ = 0.0006, *P* = 0.9807) (Additional file 3).

In this study, a history of hypertension was present in 131 patients and diabetes mellitus in 101 patients. The culture-positive rate of patients with hypertension (51.15%) was higher than that of patients without hypertension (45.98%) (χ^2^ = 1.5207, *P* = 0.4675). Furthermore, we analysed the interactive impact of hypertension and sex on culture-positivity rates. The culture-positive rate was higher among hypertensive women (58.14%, 50/81) than among hypertensive men (40%, 20/50) (χ^2^ = 5.8662, *P* = 0.0154). However, for diabetes mellitus patients, the culture-positive rate was 45.54%, which was lower than that for patients without diabetes mellitus (49.51%) (χ^2^ = 0.4297, *P* = 0.8067) (Supplement file 2).

### Correlation between the numbers of detected species and clinical factors

There was a positive correlation between the numbers of detected species and sex (*r*_s_^*^ = 0.17921, *P* = 0.0299). Male patients (1.367 ± 0.581) had more bacterial species than female patients (1.195 ± 0.478) (Table 5). Patients admitted to the hospital in summer and autumn were likely to have more bacterial strains than those in spring and winter. However, no significant correlation was found between the numbers of detected species and seasons, patient age, educational status, history of smoking and drinking, hypertension or diabetes mellitus (all *P* > 0.05).

**Table 5:** Correlation between the numbers of detected species and clinical factors

	Numbers of detected species	*r*_*s*_^*a*^	*P*
**Season**		0.01083	0.8964
Spring	1.125 ± 0.336		
Summer	1.345 ± 0.579		
Autumn	1.368 ± 0.633		
Winter	1.053 ± 0.229		
**Age, year**		−0.10577	0.2023
-40	1.154 ± 0.376		
40–65	1.348 ± 0.564		
65-	1.200 ± 0.506		
**Sex, n(%)**		0.17921	0.0299
Female	1.195 ± 0.478		
Male	1.367 ± 0.581		
**Education, n(%)**		0.10181	0.2198
No formal education or Primary	1.149 ± 0.416		
Secondary	1.350 ± 0.577		
Tertiary	1.275 ± 0.554		
**Job, n(%)**		−0.01769	0.8316
Worker	1.333 ± 0.661		
Farmer	1.200 ± 0.484		
Clerk	1.375 ± 0.500		
Unemployed	1.239 ± 0.492		
**Marriage, n(%)**		0.02598	0.7548
Married	1.208 ± 0.415		
Single or Widowed	1.276 ± 0.548		
**High Blood Pressure, n(%)**		−0.00788	0.9245
No	1.250 ± 0.490		
<10 years	1.333 ± 0.632		
≥ 10 years	1.226 ± 0.497		
**Diabetes Mellitus, n(%)**		0.00768	0.9265
No	1.257 ± 0.523		
<10 years	1.348 ± 0.573		
≥ 10 years	1.217 ± 0.518		
**Smoking, n(%)**		−0.03684	0.6578
No	1.271 ± 0.524		
<20 years	1.294 ± 0.588		
≥ 20 years	1.217 ± 0.518		
**Drinking, n(%)**		−0.00650	0.9377
No	1.272 ± 0.544		
Yes	1.227 ± 0.429		

^a^Spearman rank correlation coefficient

## Discussion

Conjunctival sac flora play a crucial role in eye diseases, such as meibomitis, microbial keratitis, and endophthalmitis, in traumatic or surgical conditions. Exploring the conjunctival sac flora in healthy people helps us understand ocular physiology function and also helps us investigate infectious disease aetiology and therapy protocols. Multiple factors, including environmental exposure, ocular trauma, age and changes in local immunity [15–17], affect the normal conjunctival microbial flora. To the best of our knowledge, our study is the first to reveal the distribution of conjunctival microbial flora of patients before ocular surgery in Harbin, which is located in the northeastern part of China. We also investigated the systemic clinical factors and environmental factors that influence the positive bacterial culture in the conjunctival sac of patients. Younger ages of male patients, older ages of patients, female patients with hypertension and patients who visited in high temperature seasons, such as summer or autumn, showed high correlations with the positive bacterial culture rate. This study provides guidance for clinicians to select more effective ocular prophylactic modalities for treatment.

In this study, the overall positive culture rate was 48.20%, which fell in the lower middle range of the previous results of 36.0–81.7% [17, 18]. Differences are likely heavily influenced by collection, transportation and culture conditions. To improve the culture positive rate, we placed the conjunctival swab samples directly into thioglycolate broth instead of 5% sheep blood or chocolate agar plates. Normal conjunctival flora is mainly comprised of coagulase-negative staphylococci (CoNS), which were also the most frequently isolated clinical organisms in our study. In our results, *Staphylococcus epidermidis* was the most frequently isolated organism (64.92%), followed by *Staphylococcus aureus* (5.76%) and *Staphylococcus xylosus* (5.24%). This finding was consistent with previously published findings showing that CoNS was the most frequently isolated ocular bacteria of patients undergoing eye surgery [19, 20] and patients with postoperative endophthalmitis [21]. Gram-negative bacteria were found in only a small portion, with *Citrobacter freundii* and *Burkholderia cepacia* being the most frequent (1.57%), and *P. aeruginosa* (1.05%) was also isolated.

Age can affect the rate of conjunctival sac flora culture in patients of different genders. In our study, the frequency of culture positivity in the conjunctiva of male patients showed an initial trend towards a reduction, and the incidence then increased with advancing age in the elderly group. The middle-aged group of 41–65 years showed the lowest positivity of bacterial isolation (40.30%). Our results were in agreement with the same type of study in Zhengzhou, which is located in the central area of China [13]. Their results showed that the rate of conjunctival bacterial isolation decreased with increasing patient age; after 41–65 years, the rates increased with age (Additional file 4). Although the trend of the conjunctival sac flora culture rate in our male patients was the same as that in the above studies, the results in our female patients were diametrically opposite. There was no turning point, and the rate of conjunctival bacterial isolation always increased with age. The young group (18–40 years) presented the lowest positivity of bacterial isolation (26.67%), and the elderly group (> 65 years) showed the highest positivity of bacterial isolation (55.41%). Age-associated changes in the normal flora of the body may be related to the elevated proinflammatory state and changed immune response in elderly people [22, 23]. The results of the study further showed that, in each age group, the positive culture rate in male patients was higher than that in female patients, especially in the younger group (≤40 years), and the difference was significant. The higher bacterial frequency in younger male patients or the elderly may have clinical implications to receive more intense prophylactic treatment.

A previous study reported that diabetes mellitus was significantly more prevalent in patients with a positive bacterial culture of the conjunctival sac than in control patients [24]. Despite this supportive evidence, conflicting views exist (Additional file 4). Martins et al. [25] found that the diabetes duration had no influence on the rate of positive cultures or the type of flora bacteria. In our results, there were no significant differences in the rates of positive cultures between the diabetic (45.54%) and nondiabetic groups (49.51%) (χ^2^ = 0.4297, *P* = 0.8067).

As is well known, climatic factors affect the morbidity of conjunctivitis around the world [26], and it is therefore worth studying whether climate affects the culture-positive rate and strain distribution of conjunctivitis. In our results, the total frequency of conjunctival bacteria in summer and autumn was surprisingly higher than that in the other seasons, which was slightly different from another study. E F Rubio [26] observed that the total frequency of conjunctival bacteria was highest in April, May, and June (spring) (Additional file 4).

Potential limitations of the present study should be mentioned. First, in this study, the traditional culture method was used, which may have skewed or compromised the growth of the cultivable microbiome from the ocular surface and missed some nonculturable microbes. Modified methods, such as genetic analysis and 16S rRNA sequencing, may lead to a much more rapid, precise and complete analysis. However, these techniques cannot distinguish viable and nonviable bacteria, and the bacteria obtained by traditional culture methods are living and the dominant bacteria. Second, because the local anaesthetic eye drops had strong antimicrobial effects, we did not use the local anaesthetic, which brought local pain to the subjects.

In conclusion, in northeastern China, coagulase-negative *Staphylococcus* was the most common preoperative conjunctival bacteria isolated*.* Hypertensive female patients, elderly patients, or patients operated on in the summer and autumn should pay more attention to perioperative treatment.

## Supplementary Information

**Additional file 1.**

**Additional file 2.**

**Additional file 3.**

**Additional file 4.**

## Data Availability

All data have been included in the manuscript. SPSS software (v20.0, SPSS Inc., Chicago, IL, USA).
