# Supplementary figures and images for: Bacteriological profile of conjunctiva bacterial Flora in Northeast China: a hospital-based study
Source: BMC Ophthalmol. 2022 May 16;22:223. doi: 10.1186/s12886-022-02441-8 (PMC9109342; doi:10.1186/s12886-022-02441-8)

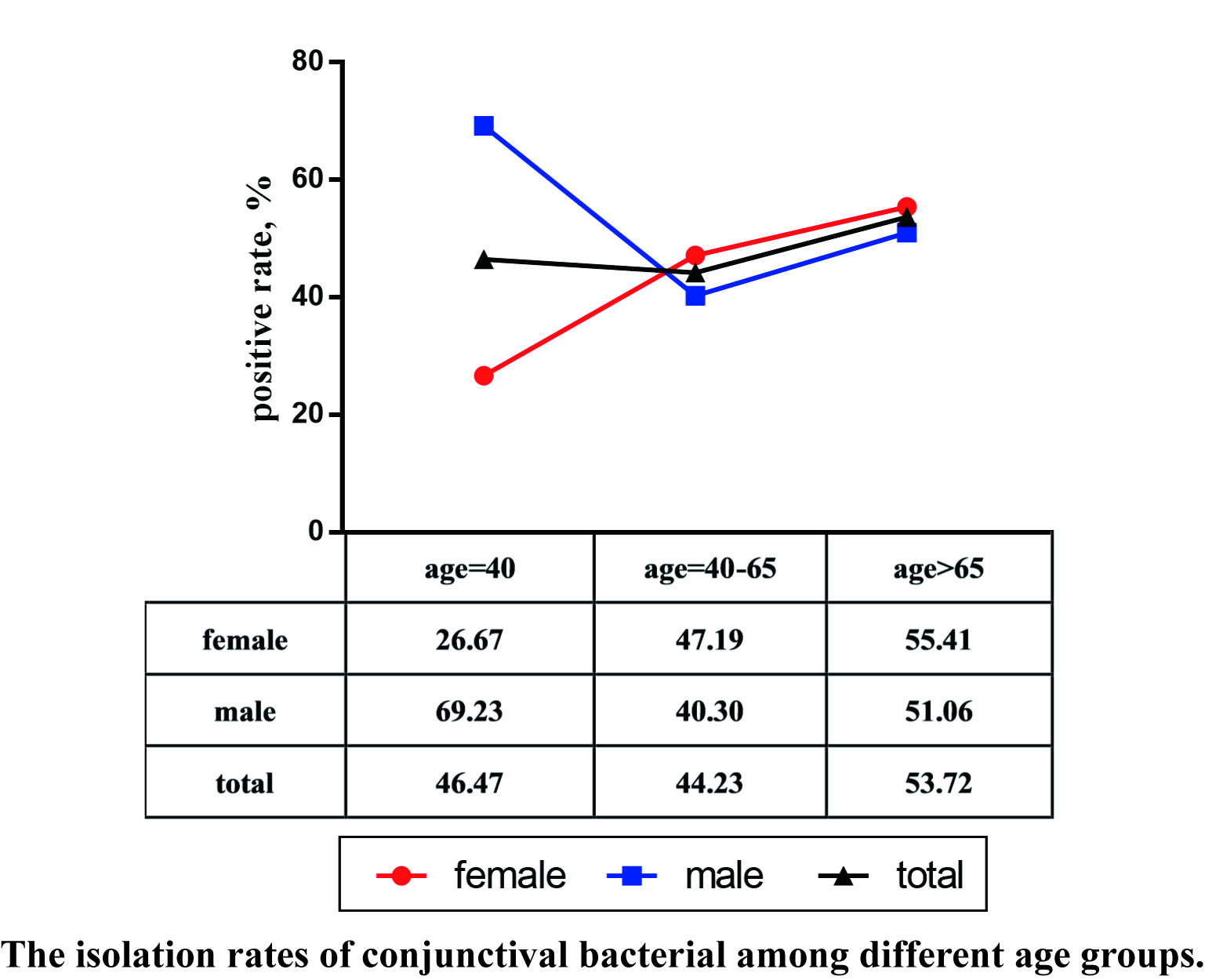

Supplement: Supplementary file 1 — Additional file 1. [file 12886_2022_2441_MOESM1_ESM.tif]
