# Supplementary material for: Bacteriological profile of conjunctiva bacterial Flora in Northeast China: a hospital-based study
Source: BMC Ophthalmol. 2022 May 16;22:223. doi: 10.1186/s12886-022-02441-8 (PMC9109342; doi:10.1186/s12886-022-02441-8)
Supplement: Supplementary file 2 — Additional file 2. [file 12886_2022_2441_MOESM2_ESM.doc]

**The isolations from conjunctival samples of every season**

| **Season** | **Culture-negative** | **Culture-positive** | ***x^2^*** | ***P*** |
| --- | --- | --- | --- | --- |
| Spring | 46(58.97) | 32(41.03) | 2.1586 | 0.1418 |
| Summer | 60(50.85) | 58(49.15) | 0.0704 | 0.7907 |
| Autumn | 29(43.28) | 38(56.72) | 2.4962 | 0.1141 |
| Winter | 23(54.76) | 19(45.24) | 0.1708 | 0.6794 |
