# Supplementary material for: Bacteriological profile of conjunctiva bacterial Flora in Northeast China: a hospital-based study
Source: BMC Ophthalmol. 2022 May 16;22:223. doi: 10.1186/s12886-022-02441-8 (PMC9109342; doi:10.1186/s12886-022-02441-8)
Supplement: Supplementary file 3 — Additional file 3. [file 12886_2022_2441_MOESM3_ESM.doc]

**Clinical factors related to positive or negative bacterial culture in the conjunctival sac of 305 patients**

| **Clinical factors** | **Culture-negative** | **Culture-positive** | **χ^2^** | ***P*** |
| --- | --- | --- | --- | --- |
| **History of smoking** |  |  | 0.8866 | 0.6419 |
| No | 123(53.48) | 107(46.52) |  |  |
| ˂20 years | 13(43.33) | 17(56.67) |  |  |
| ≥20 yesar | 22(48.89) | 23(51.11) |  |  |
| **History of drinking** |  |  | 0.0006 | 0.9807 |
| No | 134(51.75) | 125(48.26) |  |  |
| Yes | 24(52.17) | 22(47.83) |  |  |
| **Hypertension** |  |  | 1.5207 | 0.4675 |
| No | 94(54.02) | 80(45.98) |  |  |
| Yes | 64(48.85) | 67(51.15) |  |  |
| **Diabetes mellitus** |  |  | 0.4297 | 0.8067 |
| No | 103(50.49) | 101(49.51) |  |  |
| Yes | 55(54.46) | 46(45.54) |  |  |
