# Supplementary material for: Bacteriological profile of conjunctiva bacterial Flora in Northeast China: a hospital-based study
Source: BMC Ophthalmol. 2022 May 16;22:223. doi: 10.1186/s12886-022-02441-8 (PMC9109342; doi:10.1186/s12886-022-02441-8)
Supplement: Supplementary file 4 — Additional file 4. [file 12886_2022_2441_MOESM4_ESM.doc]

**Influencing factors of positive culture of conjunctival sac flora in studies from different parts of world**

|  | **Influencing factor** | **Relation** | **Studies supporting the hypothesis** |
| --- | --- | --- | --- |
| **Culture-positive rate** | Older age | Increased culture-positive rate | Tao H et al. 2017^13^ |
|  | Diabetes mellitus | Increased culture-positive rate | Kawata T et al. 2017^24^ |
|  |  | No influence on the rate of positive cultures | Martins EN et al. 2004^25^ |
|  | Climate | April, May, and June have the highest culture-positive rate | Rubio EF. 2004^26^ |
|  |  |  |  |
